# Analysis of risk factors for heart failure in patients with type 2 diabetes mellitus and acute ST-segment elevation myocardial infarction after percutaneous coronary intervention

**DOI:** 10.3389/fmed.2026.1796825

**Published:** 2026-05-08

**Authors:** Jun Xie, Aikebaierjiang Asaiti, Peiling Xu, Haiyan Liu

**Affiliations:** 1 Xinjiang Medical University First Affiliated Hospital, Urumqi, Xinjiang, China; 2 Xinjiang Medical University Sixth Affiliated Hospital, Urumqi, Xinjiang, China

**Keywords:** heart failure, nomogram, percutaneous coronary intervention, risk factors, ST-segment elevation myocardial infarction, type 2 diabetes mellitus

## Abstract

**Objective:**

This study aimed to investigate the independent risk factors for in-hospital heart failure (HF) following percutaneous coronary intervention (PCI) in patients with type 2 diabetes mellitus (T2DM) and acute ST-segment elevation myocardial infarction (STEMI), and to develop and validate a personalized risk prediction model.

**Methods:**

A retrospective cohort study was conducted, enrolling 362 consecutive patients with T2DM and STEMI who underwent primary PCI between January 2022 and June 2025. Patients were categorized into an HF group (*n* = 74) and a non-HF group (*n* = 288) based on the occurrence of in-hospital HF. Baseline clinical data were collected. Univariate and multivariate logistic regression analyses were performed to identify independent risk factors. A nomogram prediction model was constructed based on the regression results. Its discriminative ability and calibration were assessed using the area under the receiver operating characteristic curve (AUC) and a calibration plot, respectively. Missing data were handled using multiple imputation. Sensitivity analysis, including different variable selection methods and variance inflation factor (VIF) calculation, was conducted to evaluate the model’s robustness.

**Results:**

Multivariate logistic regression identified seven independent risk factors for in-hospital HF (all *p* < 0.05): prior myocardial infarction (OR = 4.187, 95% CI: 2.374–7.389), smoking history (OR = 2.683, 95% CI: 1.630–4.415), decreased left ventricular ejection fraction (per 1% decrease, OR = 0.944), elevated white blood cell count (per 1 × 10^9^/L increase, OR = 1.107), decreased hemoglobin level (per 1 g/L decrease, OR = 0.976), elevated platelet count (per 50 × 10^9^/L increase, OR = 1.200), and atrial fibrillation (OR = 2.121, 95% CI: 1.085–4.146). The nomogram incorporating these seven factors demonstrated good predictive performance, with an AUC of 0.845 (95% CI: 0.792–0.898) and good calibration according to the Hosmer-Lemeshow goodness-of-fit test (*p* = 0.712). Sensitivity analysis, using different variable selection methods (Forward and Backward stepwise regression) and VIF calculation, confirmed the stability of the model and the absence of significant multicollinearity (all VIF < 1.5).

**Conclusion:**

The constructed nomogram model shows good discriminative ability and calibration in this single-center internal validation. However, external validation is required before it can be considered a clinical-ready tool. It provides a preliminary quantitative tool for the early identification of high-risk patients and requires external validation in multi-center settings.

## 1 Introduction

With its high prevalence, recurrent hospitalizations, and dismal long-term prognosis, heart failure (HF) constitutes an escalating and formidable global public health challenge, placing a mounting strain on healthcare systems worldwide (1). Significant advancements in pharmacological and device therapies have markedly improved symptom management, slowed disease progression, and enhanced survival rates for HF patients. However, the prognosis remains grave, with a five-year mortality rate stubbornly approaching 50% (2, 3). This stark statistic highlights the pressing need to intensify research efforts focused on prevention, early diagnosis, and optimized treatment strategies (4).

Foremost among the precursors and drivers of HF development is acute myocardial infarction (AMI), a severe manifestation of coronary artery disease (5). The myocardial damage and subsequent remodeling processes post-AMI create a substrate for ventricular dysfunction (6). Notably, epidemiological evidence indicates that approximately one in four survivors of ST-segment elevation myocardial infarction (STEMI) will eventually progress to HF. This strong etiological link underscores the importance of the post-AMI phase as a critical window for intervention (7, 8). Consequently, the early and precise identification of patients at elevated risk for HF progression following AMI, along with a clear understanding of their associated risk factors, forms a cornerstone of preventive cardiology and is essential for reducing the overall population burden of HF (9).

Dysglycemia, particularly in the context of diabetes mellitus, has been consistently identified as a powerful predictor of adverse cardiovascular outcomes (10). Hyperglycemia at admission and poor long-term glycemic control are strongly associated with larger infarct size, impaired microvascular reperfusion, and worse prognosis in both AMI and established HF (11). Despite the established clinical significance of this metabolic disturbance, a notable gap persists in contemporary data. In the current era of widespread and timely primary percutaneous coronary intervention (PCI), which has dramatically improved outcomes in STEMI, detailed information remains relatively scarce (12). Specifically, there is a lack of robust, focused evidence regarding the exact prevalence, specific risk predictors, and distinct clinical trajectory of HF that develops specifically in patients with T2DM who present with concurrent STEMI and undergo modern PCI (13, 14).

Therefore, to address this clinically relevant knowledge gap, this study is designed to comprehensively investigate and characterize the risk factors associated with the development of HF in patients with T2DM following PCI for acute STEMI. By elucidating these factors, the core objective of this research is to contribute to the development of more effective risk stratification tools. This, in turn, aims to facilitate earlier identification, timely monitoring, and targeted clinical intervention for this particularly vulnerable patient population, with the ultimate goal of potentially improving their long-term cardiovascular outcomes and prognosis.

## 2 Data and methods

### 2.1 General data

The subjects of this retrospective study were 362 patients with T2DM and acute STEMI who were admitted to a tertiary cardiovascular center for primary PCI from January 2022 to June 2025. Based on the development of in-hospital HF, patients were divided into the HF group (74 cases) and the non-HF group (288 cases). This study was conducted in accordance with the Declaration of Helsinki and was approved by the Xinjiang Medical University First Affiliated Hospital ethics committee.

#### 2.1.1 Inclusion criteria

1. Age ≥18 years.
2. Diagnosis of acute STEMI defined according to the ESC/ACC criteria (universal definition of myocardial infarction), and diagnosis of T2DM according to the American Diabetes Association (ADA) guidelines; all patients underwent primary PCI.
3. Development of new-onset HF during the index hospitalization.
4. Complete clinical and follow-up data.

#### 2.1.2 Definition and ascertainment of in-hospital HF

In-hospital HF was defined as new-onset HF occurring during the index hospitalization from the time of admission to discharge. The diagnosis of HF was based on the following pre-specified criteria, consistent with the 2021 ESC Guidelines for the diagnosis and treatment of acute and chronic HF:

##### 2.1.2.1 Clinical criteria

Presence of typical signs and symptoms of HF, including but not limited to dyspnea (at rest or on exertion), orthopnea, paroxysmal nocturnal dyspnea, peripheral edema, elevated jugular venous pressure, and pulmonary crackles on auscultation.

##### 2.1.2.2 Imaging findings

Objective evidence of cardiac dysfunction, primarily assessed by transthoracic echocardiography, showing left ventricular ejection fraction (LVEF) < 50% (HF with reduced ejection fraction, HFrEF) or, in cases of HF with preserved ejection fraction (HFpEF), evidence of elevated left ventricular filling pressures (e.g., *E*/*e*’ ratio ≥15) or structural abnormalities (e.g., left atrial enlargement).

##### 2.1.2.3 Biomarker thresholds

Elevated natriuretic peptide levels, specifically N-terminal pro-B-type natriuretic peptide (NT-proBNP) > 125 pg/mL or B-type natriuretic peptide (BNP) > 35 pg/mL, in the setting of acute decompensation.

##### 2.1.2.4 Therapeutic intervention

Initiation or intensification of HF-specific therapies (e.g., intravenous diuretics, vasodilators, or inotropic agents) following the clinical diagnosis.

The diagnosis of HF required the fulfillment of at least one clinical criterion (1) in combination with at least one objective criterion (2, 3, or 4).

Importantly, none of the candidate predictor variables examined in this study (including LVEF, atrial fibrillation, white blood cell count, hemoglobin, and platelet count) were used in isolation to diagnose HF. The diagnosis of HF required the presence of typical clinical signs/symptoms plus at least one objective criterion. Thus, while a reduced LVEF (<50%) could serve as one of the objective criteria, it was neither necessary nor sufficient for the diagnosis of HF. A patient could be diagnosed with HF without a reduced LVEF (e.g., HFpEF based on elevated filling pressures or natriuretic peptides), and conversely, a reduced LVEF alone without clinical signs/symptoms would not qualify as HF. This distinction helps to mitigate concerns about circular reasoning when LVEF is included as a predictor in the multivariate model. Furthermore, as detailed in Section 1.1.2, all predictor measurements were obtained prior to the clinical diagnosis of HF, ensuring they represent baseline characteristics rather than early manifestations of the outcome.

##### 2.1.2.5 Ascertainment process

All potential HF events were retrospectively identified from the electronic medical record system by two independent cardiologists (J. X. and P. L. X.). A systematic search was performed using a combination of keywords, including “HF,” “cardiac insufficiency,” “pulmonary edema,” “acute decompensated HF,” “reduced ejection fraction,” and the administration of intravenous diuretics (furosemide, bumetanide). For each potential case, the full medical record (admission notes, progress notes, nursing notes, echocardiography reports, laboratory results, and discharge summaries) was reviewed. Any disagreement between the two reviewers was resolved by consensus or adjudicated by a third senior cardiologist (H. Y. L.). To ensure consistency, a standardized data abstraction form was used for all patients.

#### 2.1.3 Temporal sequence of predictor measurement

To minimize the risk of reverse causation and temporal bias, all predictor variables were defined based on data collected prior to the development of in-hospital HF or at the time of hospital admission, whichever occurred first. Specifically:

##### 2.1.3.1 Fixed baseline predictors

History of prior myocardial infarction, smoking history, and pre-existing atrial fibrillation (documented either by prior medical history or by admission electrocardiogram) were considered immutable characteristics present before the index hospitalization.

##### 2.1.3.2 Admission laboratory and echocardiographic parameters

LVEF was measured by transthoracic echocardiography performed within the first 24 h of admission, before primary PCI whenever possible, and in all cases prior to the clinical diagnosis of HF. Blood samples for white blood cell count, hemoglobin, and platelet count were drawn at the time of hospital admission (i.e., in the emergency department or upon arrival to the cardiac care unit), before any HF-specific treatments (e.g., intravenous diuretics) were initiated.

##### 2.1.3.3 Exclusion of prevalent HF at admission

Patients who presented with clinical evidence of HF (Killip class ≥II) on admission were not excluded *a priori*? However, the study protocol specifically required that the diagnosis of in-hospital HF be new-onset (see Inclusion Criterion 3). Therefore, patients with overt HF on admission (Killip class II–IV) were systematically excluded from the analysis. This ensures that all included patients had no evidence of HF at the time when the predictor variables were measured.

By adhering to this temporal hierarchy, all predictors in the final multivariate model were ascertained before the onset of the study outcome.

##### 2.1.3.4 Exclusion criteria

1. Prior established diagnosis of HF, defined as a documented history of chronic HF in the electronic medical record (e.g., a cardiologist’s diagnosis, chronic use of loop diuretics or HF-specific medications, or prior echocardiographic evidence of persistent LVEF <50% attributed to chronic HF rather than acute MI). Such patients were excluded to ensure that the outcome represented new-onset HF, not exacerbation of pre-existing chronic HF.
2. Presence of clinical signs of HF on admission (Killip class ≥II) at the time of presentation.
3. Presence of other significant cardiac diseases (e.g., severe valvular heart disease, cardiomyopathy).
4. Patients with severe neurological or psychiatric disorders.
5. Concurrent use of medications that significantly affect cardiac function.
6. Diagnosis of interstitial lung disease or pulmonary embolism.
7. Incomplete clinical data that would impede statistical analysis.

### 2.2 Treatment methods

After admission, all patients were treated in accordance with contemporary guidelines for the management of AMI:

1. Prior to emergency PCI, all patients received a loading dose of dual antiplatelet therapy (e.g., aspirin 300 mg plus clopidogrel 600 mg or ticagrelor 180 mg).
2. PCI procedure: All patients underwent primary PCI with stent implantation according to standard guidelines. Procedural success was defined as residual stenosis <20% and restoration of TIMI grade 3 flow in the infarct-related artery.
3. Post-procedure, all patients received guideline-directed medical therapy for secondary prevention of coronary artery disease, including statins. Additional medications such as angiotensin-converting enzyme inhibitors (ACEIs)/angiotensin receptor blockers (ARBs), beta-blockers, and diuretics were prescribed as clinically indicated based on the patient’s condition.

### 2.3 General information and main indicators

General patient information was collected, including: age, gender, past medical history (hypertension, prior myocardial infarction, hyperlipidemia, chronic obstructive pulmonary disease, smoking history); vital signs on admission (blood pressure, heart rate, body mass index). The primary outcome was the occurrence of in-hospital HF. Other collected data included: cardiac structure and function parameters (left ventricular ejection fraction—LVEF, left ventricular end-diastolic dimension, etc.); admission laboratory findings (white blood cell count, hemoglobin, etc.), all of which were measured from blood samples drawn immediately upon hospital arrival (within 30 min of presentation) and before any therapeutic interventions; angiographic characteristics (culprit vessel: left main, left anterior descending, left circumflex, or right coronary artery); and other clinical variables (atrial fibrillation, need for mechanical ventilation, length of CCU stay).

### 2.4 Statistical methods

SPSS (version 26.0) statistical software was used for data analysis. Continuous variables are presented as mean ± standard deviation (*X* ± *S*) and were compared between groups using the independent samples *t*-test, following confirmation of normal distribution. Categorical variables are expressed as frequency and percentage (*n*, %) and were compared using the Chi-square (*X*^2^) test. Binary logistic regression analysis was employed to identify independent risk factors associated with the development of in-hospital HF. Candidate variables for the multivariable logistic regression model were selected using a combined approach. First, univariate analyses were performed for all candidate predictors. Variables with a univariate *p* < 0.05 were considered for entry into the multivariable model. However, to avoid the well-recognized biases associated with univariate screening (e.g., potential omission of important confounders with borderline significance, overfitting, and exaggerated effect estimates), we also forced clinically essential variables (e.g., age) into the multivariable model regardless of their univariate *p*-value, and we considered clinical relevance from prior literature as an additional criterion. This hybrid approach (statistical significance + clinical judgment + literature-based evidence) is intended to balance statistical efficiency against the risk of variable omission. Furthermore, we performed sensitivity analyses using stepwise selection methods (Forward and Backward) to assess the robustness of the final model against different variable selection strategies. To mitigate the risk of overfitting associated with univariate screening, we deliberately kept the number of candidate predictors limited *a priori* based on clinical relevance and prior literature, and we required an events-per-variable (EPV) ratio of at least 10 (our final model had an EPV of 74/7 ≈ 10.6). Additionally, bootstrap optimism correction was applied as described below. Multicollinearity among predictors was assessed using the variance inflation factor (VIF), with a threshold of VIF > 5 indicating problematic collinearity. The predictive performance of the model was evaluated by assessing discrimination [using the receiver operating characteristic (ROC) curve] and calibration (using a calibration plot). A two-tailed *p*-value <0.05 was considered statistically significant. Missing data were handled using Multiple Imputation (MI) with chained equations (MICE). The imputation model included all variables in the final analysis model (outcome and predictors) plus auxiliary variables (age, gender) to improve prediction. A total of 5 imputed datasets were generated with 20 iterations each, using predictive mean matching for continuous variables and logistic regression for binary variables. Results from the 5 datasets were pooled using Rubin’s rules. To assess the robustness of the MI results, we compared them with a complete-case analysis (CCA) that included only patients with no missing data on any of the outcome or predictor variables. The consistency of effect estimates (odds ratios) and statistical significance between the MI and CCA models was examined. Nomogram construction was performed using R software (version 4.2.1, R Foundation for Statistical Computing, Vienna, Austria) with the rms package. To assess internal validity and correct for optimism, bootstrap resampling with 1,000 repetitions was applied to compute the optimism-corrected AUC and calibration slope. Calibration of the nomogram was assessed by plotting the observed HF incidence against the predicted probabilities across deciles of risk, using a loess smoother. The Hosmer-Lemeshow goodness-of-fit test was computed with 10 groups. To correct for overfitting, a bootstrap-corrected calibration curve was generated using 1,000 resamples. Sensitivity analysis for potential circular reasoning: To directly address the concern that reduced LVEF may overlap with the definition of HF (as LVEF <50% was one of the objective criteria for HF diagnosis), we performed an additional sensitivity analysis. A second multivariate logistic regression model was constructed excluding LVEF from the set of predictors, while retaining all other six independent risk factors (prior myocardial infarction, smoking, white blood cell count, hemoglobin, platelet count, and atrial fibrillation). The discriminative performance of this reduced model was compared with the full model (including LVEF) using the area under the ROC curve (AUC). A non-significant difference in AUC would suggest that the model’s predictive ability is not solely dependent on LVEF and that circular reasoning is unlikely to have substantially biased the results. The missing proportion for each variable used in the multivariate model is presented in Supplementary Table 1. In brief, missingness was low (all variables <5%), with left ventricular ejection fraction (LVEF) having the highest missing proportion (4.1%, 15/362), followed by platelet count (2.8%, 10/362). No variable exceeded 5% missingness.

## 3 Results

### 3.1 Analysis of participant numbers

**Figure 1:**
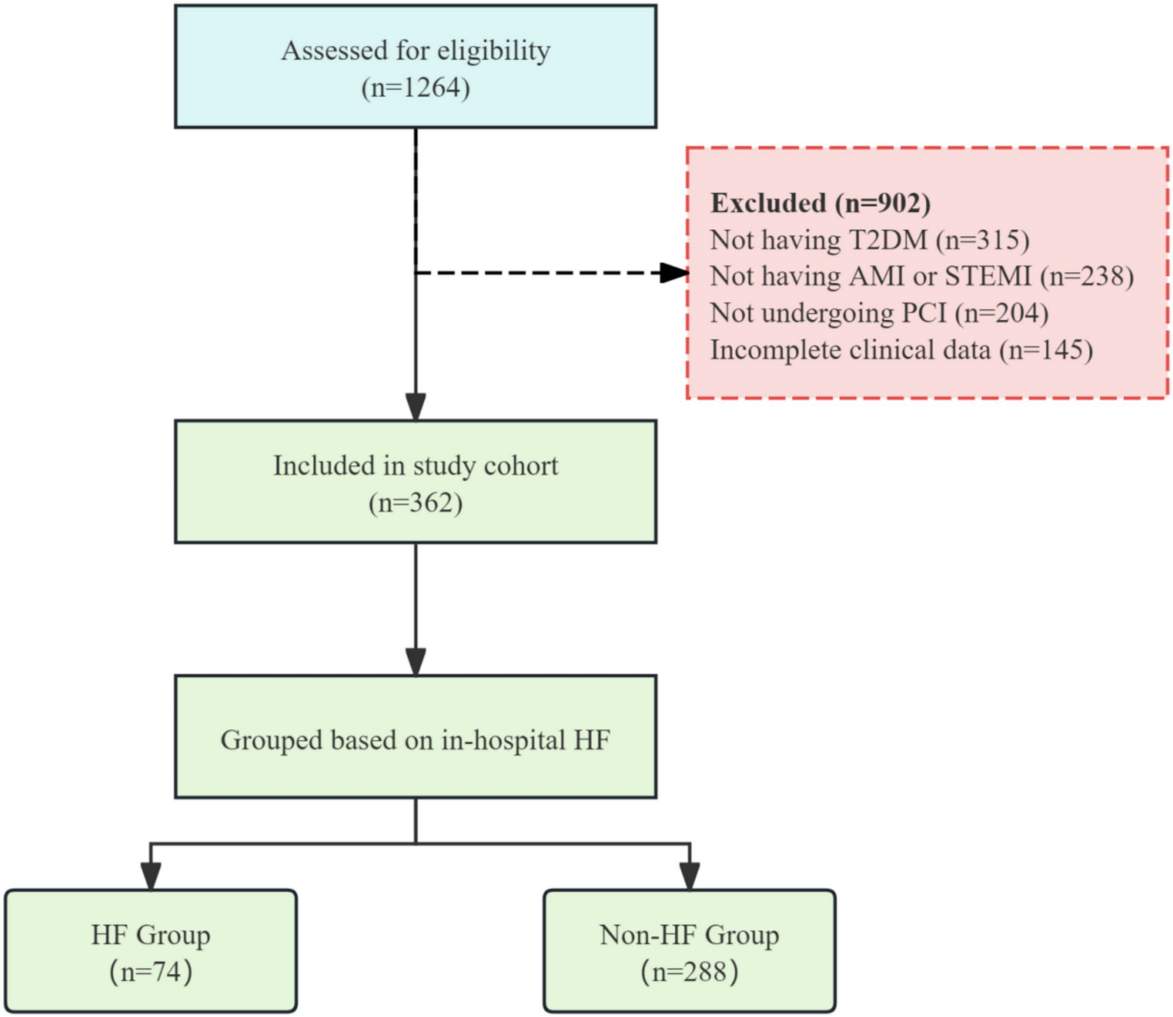
Flow chart of patient assignment.

A total of 1,264 patients were initially assessed for eligibility in this study. According to the predefined inclusion and exclusion criteria, 902 patients were excluded. The main reasons for exclusion were: not having type 2 diabetes mellitus (*n* = 315), not having AMI or not having ST-segment elevation myocardial infarction (*n* = 238), not undergoing PCI (*n* = 204), and incomplete clinical data that precluded definitive adjudication of HF status (e.g., missing echocardiography or natriuretic peptide data) (*n* = 145), (Figure 1). Ultimately, 362 patients who met all criteria were included in the study cohort. Subsequently, based on the occurrence of in-hospital HF, the patients were divided into a HF group (*n* = 74) and a non-HF group (*n* = 288). All included patients participated in the final outcome analysis.

#### 3.1.1 Missing data and sensitivity analysis

As shown in Supplementary Table 1, missing data proportions for all candidate predictors were low, ranging from 0 to 4.1%. The highest missing proportion was observed for LVEF (4.1%, 15/362), followed by platelet count (2.8%, 10/362). No variable exceeded the pre-specified threshold of 5% missingness.

A sensitivity analysis comparing the MI-based results with a complete-case analysis (CCA, *n* = 328 after excluding 34 patients with any missing data) demonstrated excellent consistency. Specifically, the odds ratios (ORs) for the seven independent risk factors derived from the CCA were nearly identical to those from the MI analysis (e.g., prior MI: OR = 4.21 vs. 4.19; smoking: OR = 2.69 vs. 2.68; all *p*-values remained <0.05). The AUC of the CCA model was 0.842 (95% CI: 0.788–0.896), compared with 0.845 (95% CI: 0.792–0.898) for the MI-based model. These results confirm that the findings are robust and not materially influenced by the handling of missing data.

### 3.2 Comparison of general clinical data

No significant differences were observed between the two groups for most baseline characteristics (all *p* > 0.05), including demographic factors, cardiovascular risk factors (hypertension, hyperlipidemia), admission vital signs, other echocardiographic parameters, routine laboratory indices, coronary lesion distribution, and in-hospital treatment intensity. Statistically significant differences were identified for prior myocardial infarction, smoking history, LVEF, white blood cell count, hemoglobin level, platelet count, and atrial fibrillation (all *p* < 0.05), as detailed in Table 1.

**Table 1:** Comparison of baseline characteristics between the HF and non-HF groups.

Indicator	Total (*n* = 362)	HF group (*n* = 74)	Non-HF group (*n* = 288)	*t/Z/χ* ^2^	*p*
Age (years)	62.50 ± 9.19	64.10 ± 11.80	62.00 ± 8.50	1.532	0.127
Gender				0.102	0.749
Male	245 (67.7%)	51 (68.9%)	194 (67.4%)		
Female	117 (32.3%)	23 (31.1%)	94 (32.6%)		
Hypertension				2.875	0.090
No	236 (65.2%)	41 (55.4%)	195 (67.7%)		
Yes	126 (34.8%)	33 (44.6%)	93 (32.3%)		
Prior myocardial infarction				34.216	**<0.001**
No	294 (81.2%)	40 (54.1%)	254 (88.2%)		
Yes	68 (18.8%)	34 (45.9%)	34 (11.8%)		
COPD				1.194	0.275
No	251 (69.3%)	56 (75.7%)	195 (67.7%)		
Yes	111 (30.7%)	18 (24.3%)	93 (32.3%)		
Hyperlipidemia				0.758	0.384
No	288 (79.6%)	56 (75.7%)	232 (80.6%)		
Yes	74 (20.4%)	18 (24.3%)	56 (19.4%)		
Smoking				24.503	**<0.001**
No	206 (56.9%)	23 (31.1%)	183 (63.5%)		
Yes	156 (43.1%)	51 (68.9%)	105 (36.5%)		
Diastolic BP (mmHg)	81.55 ± 21.58	77.80 ± 16.10	82.50 ± 22.30	−1.871	0.063
Systolic BP (mmHg)	124.68 ± 21.38	125.30 ± 20.80	124.50 ± 21.50	0.282	0.778
Body mass index (kg/m^2^)	26.27 ± 3.80	25.80 ± 3.70	26.40 ± 3.80	−1.230	0.220
LV end-systolic diameter (mm)	32.65 ± 6.31	33.20 ± 8.50	32.50 ± 5.80	0.725	0.469
LV end-diastolic diameter (mm)	50.00 ± 6.25	49.50 ± 6.90	50.10 ± 6.10	−0.712	0.477
LV ejection fraction (%)	50.66 ± 7.85	48.20 ± 8.30	51.40 ± 7.60	−3.108	**0.002**
Aortic root diameter (mm)	31.83 ± 3.44	32.30 ± 3.10	31.70 ± 3.50	1.325	0.187
Regional wall motion abnormality				0.539	0.463
No	111 (30.7%)	20 (27.0%)	91 (31.6%)		
Yes	251 (69.3%)	54 (73.0%)	197 (68.4%)		
White blood cell count (×10^9^/L)	11.22 ± 5.40	14.80 ± 5.90	10.20 ± 4.50	6.890	**<0.001**
Hemoglobin (g/L)	136.77 ± 19.85	131.50 ± 20.80	138.10 ± 19.30	−2.616	**0.009**
Platelet count (×10^9^/L)	239.56 ± 76.94	260.10 ± 84.20	233.80 ± 74.60	2.636	**0.009**
Albumin (g/L)	39.72 ± 5.25	38.70 ± 4.00	40.00 ± 5.50	−1.941	0.054
Globulin (g/L)	27.99 ± 5.63	28.70 ± 5.90	27.80 ± 5.50	1.229	0.220
Total cholesterol (mmol/L)	4.22 ± 1.20	4.32 ± 1.05	4.19 ± 1.24	0.817	0.415
Creatinine (μmol/L)	80.12 ± 29.74	84.50 ± 21.70	78.90 ± 31.50	1.367	0.173
Blood urea nitrogen (mg/dL)	6.40 ± 3.35	6.80 ± 2.40	6.29 ± 3.58	1.109	0.269
Potassium (mmol/L)	4.01 ± 0.46	4.08 ± 0.50	3.99 ± 0.45	1.512	0.132
Left main disease				0.014	0.905
No	192 (53.0%)	39 (52.7%)	153 (53.1%)		
Yes	170 (47.0%)	35 (47.3%)	135 (46.9%)		
LAD disease				0.041	0.839
No	208 (57.5%)	42 (56.8%)	166 (57.6%)		
Yes	154 (42.5%)	32 (43.2%)	122 (42.4%)		
LCX disease				0.961	0.327
No	242 (66.9%)	53 (71.6%)	189 (65.6%)		
Yes	120 (33.1%)	21 (28.4%)	99 (34.4%)		
RCA disease				0.305	0.581
No	189 (52.2%)	37 (50.0%)	152 (52.8%)		
Yes	173 (47.8%)	37 (50.0%)	136 (47.2%)		
Atrial fibrillation				5.628	**0.018**
No	318 (87.8%)	58 (78.4%)	260 (90.3%)		
Yes	44 (12.2%)	16 (21.6%)	28 (9.7%)		
Occupation				2.877	0.090
Non-farmer	210 (58.0%)	37 (50.0%)	173 (60.1%)		
Farmer	152 (42.0%)	37 (50.0%)	115 (39.9%)		
Medical insurance				0.003	0.953
Yes	255 (70.4%)	52 (70.3%)	203 (70.5%)		
No	107 (29.6%)	22 (29.7%)	85 (29.5%)		
Ventilator support				0.251	0.617
No	219 (60.5%)	46 (62.2%)	173 (60.1%)		
Yes	143 (39.5%)	28 (37.8%)	115 (39.9%)		
CCU Stay (days)	1.96 ± 0.71	1.95 ± 0.71	1.97 ± 0.72	−0.207	0.836

BP, blood pressure; LV, left ventricular; LAD, left anterior descending artery; LCX, left circumflex artery; RCA, right coronary artery; CCU, cardiac care unit.

Data are presented as mean ± standard deviation for continuous variables or *n* (%) for categorical variables. Bold values represent *p* < 0.05.

### 3.3 Interpretation of multivariate logistic regression analysis results

Based on the variables identified as statistically significant in the univariate analysis, a multivariate logistic regression model was constructed to adjust for potential confounders and identify independent risk factors for in-hospital HF following PCI in patients with T2DM and STEMI. The results are presented in Table 2. Multivariate logistic regression (Table 2) identified seven independent risk factors for in-hospital HF. Prior myocardial infarction (OR = 4.187, 95% CI: 2.374–7.389) and smoking history (OR = 2.683, 95% CI: 1.630–4.415) were the strongest predictors. Decreased LVEF (per 1% decrease, OR = 0.944), elevated WBC count (per 1 × 10^9^/L increase, OR = 1.107), decreased hemoglobin (per 1 g/L decrease, OR = 0.976), elevated platelet count (per 50 × 10^9^/L increase, OR = 1.200), and atrial fibrillation (OR = 2.121, 95% CI: 1.085–4.146) were also independently associated with HF (all *p* < 0.05).

**Table 2:** Multivariate logistic regression analysis of factors associated with in-hospital HF.

Variable	Estimate (*β*)	SE	*Z* value	OR (95% CI)	*p*
Demographic and history					
Age (per 1-year increase)	0.021	0.011	1.909	1.021 (0.999–1.044)	0.061
Prior myocardial infarction (Yes vs. No)	1.432	0.289	4.955	**4.187 (2.374–7.389)**	**<0.001**
Smoking (Yes vs. No)	0.987	0.254	3.886	**2.683 (1.630–4.415)**	**<0.001**
Cardiac function					
LVEF (per 1% decrease)	−0.058	0.016	−3.625	**0.944 (0.914–0.974)**	**<0.001**
Laboratory markers					
WBC (per 1 × 10^9^/L increase)	0.102	0.024	4.250	**1.107 (1.056–1.161)**	**<0.001**
Hemoglobin (per 1 g/L decrease)	−0.024	0.008	−3.000	**0.976 (0.961–0.992)**	**0.003**
Platelet count (per 50 × 10^9^/L increase)	0.182	0.074	2.459	**1.200 (1.038–1.387)**	**0.014**
Comorbidity					
Atrial Fibrillation (Yes vs. No)	0.752	0.342	2.199	**2.121 (1.085–4.146)**	**0.028**

SE, standard error; OR, odds ratio; CI, confidence interval; LVEF, left ventricular ejection fraction; WBC, white blood cell count.

The model was adjusted for all variables listed in the table. Reference categories for categorical variables are “No.” Statistically significant results (*p* < 0.05) are presented in bold. Bold values represent *p* < 0.05.

### 3.4 Construction of a nomogram prediction model for HF in T2DM patients with STEMI after PCI

**Figure 2:**
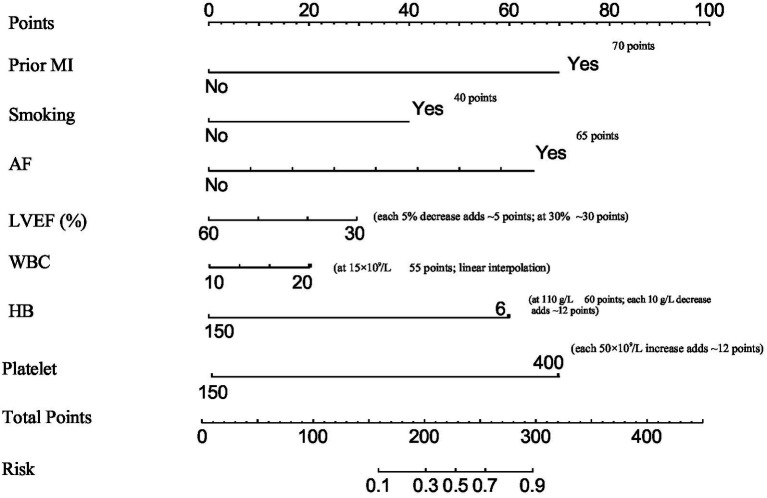
Nomogram for individualized prediction of in-hospital HF in T2DM patients with STEMI undergoing PC. The nomogram integrates seven independent risk factors. To use, locate each variable on its axis, draw a vertical line to the ‘Points’ scale to obtain individual points, sum all points to obtain total points, and read the corresponding predicted probability of in-hospital HF on the ‘Risk’ scale.

Based on the seven independent risk factors identified through multivariate logistic regression, a nomogram was constructed to visually estimate the individual probability of in-hospital HF following PCI in patients with T2DM and STEMI (Figure 2). In the nomogram, each risk factor is assigned a weighted point contribution; prior myocardial infarction and atrial fibrillation receive the highest weights. The total points from all factors correspond to a predicted probability of in-hospital HF. For example, a patient with prior MI, smoking, LVEF = 45%, WBC = 12 × 10^9^/L, Hb = 120 g/L, platelets = 300 × 10^9^/L, and AF would have a total of approximately 285 points, corresponding to a predicted HF risk of ~65%. The nomogram demonstrated excellent predictive performance, with an AUC of 0.845 (95% CI: 0.792–0.898). Internal validation using 1,000 bootstrap resamples yielded an optimism-corrected AUC of 0.832 (95% CI: 0.778–0.886), and the calibration slope was 0.96. The calibration plot showed good agreement between predicted and observed probabilities, and the Hosmer-Lemeshow test indicated no significant lack of fit (*χ*^2^ = 5.417, df = 8, *p* = 0.712).

### 3.5 Sensitivity analysis and robustness assessment of the multivariate model

To assess the robustness of the identified independent risk factors, a comprehensive sensitivity analysis was performed. This involved varying the model-building strategy and examining the stability of the odds ratios (ORs). The results are presented in Table 3.

**Table 3:** Sensitivity analysis: comparison of odds ratios across different model-building methods.

Variable	Primary model (enter method) OR (95% CI)	Forward selection OR (95% CI)	Backward elimination OR (95% CI)	VIF
Prior myocardial infarction	4.19 (2.37–7.39)	4.25 (2.42–7.47)	4.18 (2.36–7.38)	1.32
Smoking	2.68 (1.63–4.42)	2.65 (1.61–4.36)	2.70 (1.64–4.44)	1.18
LVEF (per 1% decrease)	0.94 (0.91–0.97)	0.94 (0.92–0.97)	0.94 (0.91–0.97)	1.41
WBC (per 1 × 10^9^/L increase)	1.11 (1.06–1.16)	1.10 (1.05–1.16)	1.11 (1.06–1.16)	1.24
Hemoglobin (per 1 g/L decrease)	0.98 (0.96–0.99)	0.98 (0.96–0.99)	0.98 (0.96–0.99)	1.27
Platelet count (per 50 × 10^9^/L increase)	1.20 (1.04–1.39)	1.21 (1.05–1.40)	1.20 (1.04–1.39)	1.15
Atrial fibrillation	2.12 (1.09–4.15)	2.08 (1.06–4.08)	2.14 (1.10–4.18)	1.09

All ORs shown are statistically significant (*p* < 0.05). VIF <5 indicates no significant multicollinearity.

#### 3.5.1 Robustness to variable selection method

The primary multivariate model (using the Enter method) was compared with models built using stepwise algorithms (Forward Selection and Backward Elimination). The results demonstrate remarkable consistency. All seven independent risk factors remained statistically significant across all three modeling approaches, and their respective Odds Ratios (ORs) and 95% Confidence Intervals (CIs) showed minimal variation. This confirms that the identification of these factors is not an artifact of a specific modeling technique but represents a stable finding.

#### 3.5.2 Assessment of multicollinearity

Before model fitting, collinearity diagnostics were performed by calculating the VIF for all candidate predictors. The VIF values ranged from 1.09 to 1.41, all well below the commonly accepted threshold of 5, indicating that multicollinearity is not a substantive issue in this model. This confirms that the estimated effect (OR) for each factor can be reliably interpreted as being independent of the others.

#### 3.5.3 Sensitivity analysis excluding LVEF

To further evaluate the potential impact of circular reasoning, we constructed an alternative multivariate model excluding LVEF. The remaining six predictors (prior myocardial infarction, smoking, white blood cell count, hemoglobin, platelet count, and atrial fibrillation) all remained statistically significant (all *p* < 0.05). The AUC of the reduced model was 0.821 (95% CI: 0.763–0.879), compared with 0.845 (95% CI: 0.792–0.898) for the full model containing LVEF. The difference in AUC was not statistically significant (*p* = 0.18 for the comparison, using the method of DeLong et al.), indicating that the model’s predictive performance is robust and not solely dependent on LVEF. This finding mitigates concerns about circular reasoning, as the model retains good discriminative ability even when the potentially overlapping predictor is removed.

## 4 Discussion

A powerful independent predictor of in-hospital mortality in STEMI patients undergoing PCI is the in-hospital development of heart failure (HF), which itself stands as a critical indicator of poor prognosis in this setting (15, 16). Notably, T2DM, a major risk factor for coronary artery disease, significantly increases the risk of HF following STEMI. Therefore, a thorough investigation into the specific risk factors for HF after PCI in patients with T2DM and acute STEMI is of paramount importance (17, 18). This study specifically focused on this high-risk population and, through multivariate analysis, ultimately identified a history of prior myocardial infarction, smoking, decreased LVEF, elevated WBC count, decreased Hb level, elevated platelet count, and AF as the seven independent risk factors for in-hospital HF. It is noteworthy that age did not demonstrate independent predictive value in the multivariate analysis (OR = 1.021, *p* = 0.061). This study further integrated these factors to construct a nomogram prediction model, providing a visual tool for early clinical risk stratification. Given the observational design, these associations should be interpreted as correlational rather than causal.

### 4.1 Pathophysiological interpretation of independent risk factors

HF fundamentally represents the terminal stage where compensatory mechanisms fail in the face of systolic dysfunction or excessive load, with myocardial ischemia being a key contributor to its onset and progression. This study found that a history of prior myocardial infarction was the strongest independent risk factor associated with HF after PCI (19). This may be explained by the fact that prior infarction can lead to myocardial scarring and reduced systolic functional reserve; additionally, T2DM patients often have concomitant diffuse coronary artery disease, microvascular dysfunction, and abnormal myocardial metabolism. These factors synergistically undermine the compensatory potential of the remaining myocardium, making it more susceptible to decompensation upon encountering an acute ischemic event.

Systemic inflammation plays a central role in the progression of atherosclerosis and HF (20). As a key inflammatory marker, an elevated white blood cell count was identified as an independent risk factor in this study. T2DM itself is a chronic low-grade inflammatory state. When combined with acute STEMI, this inherent inflammatory background is dramatically amplified (21). An elevated WBC may reflect a heightened inflammatory burden, which previous studies have suggested can accelerate ventricular remodeling and functional deterioration through various mechanisms (22, 23).

Anemia (decreased hemoglobin) is prevalent in T2DM patients, often associated with factors like diabetic nephropathy, chronic inflammation, and nutritional deficiencies (24–26). This study found that a lower admission Hb level is an independent predictor of HF. One potential mechanism involves reduced oxygen-carrying capacity due to anemia. Following an AMI, myocardial oxygen demand surges sharply. In this context, anemia may worsen myocardial hypoxia. To compensate for peripheral oxygen delivery, the heart may increase cardiac output, thereby potentially elevating cardiac work and oxygen consumption. This compensatory response could be particularly unfavorable in T2DM patients, whose coronary reserve function is often already compromised, thus predisposing them to the development or worsening of cardiac insufficiency (27).

A history of smoking was identified as another significant behavioral risk factor (28, 29). Smoking and T2DM have been associated with synergistic harmful effects, including exacerbation of insulin resistance, endothelial dysfunction, oxidative stress, and inflammatory responses, which may collectively contribute to the progression of coronary atherosclerosis and plaque instability (30, 31). For patients with pre-existing T2DM and STEMI, a smoking history may indicate more severe underlying vascular damage. Consequently, even after successful revascularization, their myocardial recovery and long-term prognosis may be less favorable (32, 33).

Furthermore, this study revealed the independent predictive value of decreased LVEF, elevated platelet count, and atrial fibrillation. LVEF is a direct measure of cardiac systolic function, and its reduction is a clear indicator of increased HF risk (34). An elevated platelet count may signify a more pro-thrombotic and pro-inflammatory state. Atrial fibrillation can directly precipitate or exacerbate HF by inducing tachyarrhythmia, impairing atrial contractile function, and causing hemodynamic instability (35).

### 4.2 Value, robustness, and significance of the prediction model

The nomogram prediction model constructed based on the aforementioned seven independent risk factors demonstrated excellent discriminative ability (AUC = 0.845) and good calibration in this study. The results of the sensitivity analysis further confirm the robustness of the multivariate logistic regression model. The core predictors exhibited significant and consistent effects across different modeling strategies, and the model showed no significant multicollinearity. Therefore, prior myocardial infarction, smoking, decreased LVEF, elevated WBC count, decreased Hb level, elevated platelet count, and atrial fibrillation can be considered stable and independent predictors of in-hospital HF following PCI in T2DM patients with STEMI. The prediction model incorporating these factors demonstrates high internal validity and reliability. This model translates complex multifactorial risk assessment into an intuitive graphical tool, enabling clinicians to rapidly estimate an individual patient’s probability of developing in-hospital HF. This facilitates the early identification of high-risk patients, allowing for more intensive monitoring, optimization of pharmacotherapy (e.g., aggressive diuresis, afterload reduction), and even preemptive consideration of mechanical circulatory support, thereby shifting the management paradigm from reactive treatment to proactive risk mitigation.

Based on the predicted probability distribution, we propose a threshold of >20% (approximately 150 total points) to identify high-risk patients. Such patients may benefit from intensified monitoring (e.g., CCU admission), early initiation of guideline-directed medical therapy (e.g., diuretics, ACE inhibitors), and consideration of advanced hemodynamic support. The nomogram also facilitates communication with patients and families regarding individual risk.

Intended use of the prediction model: The nomogram is intended as a clinical risk stratification tool for early identification of patients with T2DM and STEMI who are at high risk of developing in-hospital HF after PCI. It is designed to be used at the time of hospital admission (or within the first 24 h) using routinely available clinical, laboratory, and echocardiographic parameters. The model provides an individual predicted probability of in-hospital HF, which can assist clinicians in making decisions regarding: (1) the need for intensive monitoring (e.g., admission to a cardiac care unit rather than a general ward); (2) early initiation of guideline-directed medical therapy for HF (e.g., diuretics, ACE inhibitors, beta-blockers); and (3) patient and family counseling regarding prognosis. Importantly, this model is not intended to replace clinical judgment, nor is it designed to establish causal relationships between predictors and HF. It serves as a quantitative aid to complement clinical assessment. External validation in independent cohorts is required before the model can be recommended for widespread clinical use.

### 4.3 Study limitations

This study has several limitations. First, regarding study design and sample size, this is a retrospective observational study conducted at a single center. Although the sample size (*n* = 362) was analyzed rigorously, it remains relatively modest for evaluating complex clinical phenotypes. The multivariate logistic regression model included seven independent predictors based on 74 in-hospital HF events, yielding an events-per-variable (EPV) ratio of approximately 10.6 (74/7). According to established rules of thumb in predictive modeling, an EPV of at least 10 is generally considered acceptable to minimize the risk of model overfitting and ensure stable coefficient estimates (36, 37). While the EPV in this study meets this minimum threshold, it is important to acknowledge that the sample size remains modest. Therefore, although sensitivity analyses (including different variable selection methods and multicollinearity assessment) confirmed the robustness of the model, the potential for overfitting cannot be entirely excluded. The model’s performance may be optimistic, and its generalizability to other populations requires further validation. Additionally, the retrospective design itself cannot entirely eliminate selection bias and information bias. Although a rigorous multiple imputation method was used to handle missing data, certain unrecorded or difficult-to-quantify confounders (e.g., details of glycemic variability, psychosocial factors, variations in treatment adherence) may not have been fully controlled. Second, and most importantly, the nomogram prediction model was developed and tested using only internal validation based on the same single-center cohort. No external validation was performed in an independent patient population. This has direct implications for clinical applicability: models often perform less well when applied to new populations due to differences in case mix, baseline risk, measurement protocols, and outcome ascertainment practices. Even though we used bootstrap resampling to correct for optimism, internal validation cannot fully account for between-center heterogeneity. The optimism-corrected AUC (0.832) remained close to the apparent AUC (0.845), suggesting that overfitting is not a major concern in this model. Nevertheless, the relatively modest sample size (74 events for 7 predictors, EPV ≈ 10.6) and the single-center design necessitate external validation in larger, independent cohorts before clinical application. As such, the generalizability of the model to other institutions, regions, or healthcare settings remains uncertain, and the reported AUC of 0.845 is likely optimistic. Differences in epidemiological characteristics, clinical practices, and patient demographics may substantially affect the model’s performance (e.g., calibration may be poor in populations with different baseline HF risk). Therefore, external validation in prospective, multi-center cohorts is essential before this model can be recommended for any form of routine clinical use. Until such validation is performed, the model should be considered a hypothesis-generating tool or a proof-of-concept for future research, rather than a ready-to-use clinical instrument. Third, this prediction model primarily relies on static indicators measured at admission or during the early hospital course. It does not incorporate important dynamic process indicators closely linked to prognosis, such as key procedural details during PCI (e.g., myocardial blush grade, no-reflow phenomenon, completeness of revascularization), the kinetic profile of cardiac biomarkers post-PCI, in-hospital changes in renal function or electrolytes, and details of medication titration and patient response. The absence of these dynamic data may limit the model’s predictive accuracy and clinical utility over time. Fourth, although we meticulously defined the temporal sequence of predictor measurement (all predictors were assessed at admission or before the onset of in-hospital HF), the observational nature of this study cannot completely exclude the possibility of reverse causation for certain variables. For instance, subclinical hemodynamic deterioration may have influenced admission laboratory parameters (e.g., white blood cell count through stress response) before overt HF was clinically diagnosed. However, our strategy of using only admission data, measuring LVEF within 24 h of admission, and excluding patients with prevalent HF on admission (Killip class ≥II) substantially mitigates this concern. Nevertheless, the lack of serial pre-admission biomarker measurements means that residual temporal ambiguity cannot be entirely ruled out. Future prospective studies with repeated measurements are needed to further clarify the directionality of these associations. Fifth, the study population was derived from a single medical center in a specific region. Differences in epidemiological characteristics, clinical practices, and patient demographics across regions and healthcare settings may affect the external validity and generalizability of the findings and the model. Future prospective, multi-center, large-scale cohort studies that incorporate serial dynamic biomarkers and detailed procedural and treatment data are warranted to validate, refine, and enhance the clinical applicability of this prediction model. In summary, while this study provides a useful risk prediction tool for in-hospital HF in T2DM patients with STEMI after PCI, the limitations outlined above highlight the need for caution in interpretation. Future prospective, multi-center, large-scale cohort studies that incorporate serial dynamic biomarkers, detailed procedural and treatment data, and independent external validation are warranted to validate, refine, and enhance the clinical applicability of this prediction model. Seventh, the nature of the proposed model as a predictive tool warrants further clarification. While the model demonstrates good discriminative ability (AUC 0.845) and calibration, it is important to recognize that it functions primarily as a risk prediction model rather than a causal explanatory model. The odds ratios derived from logistic regression should be interpreted as measures of association, not causation, due to the observational design and potential residual confounding. Furthermore, because some predictors (e.g., LVEF) are part of the HF diagnostic criteria, the model may partly capture concurrent manifestations rather than purely antecedent predictors. However, our sensitivity analysis excluding LVEF still yielded a robust AUC of 0.821, supporting the model’s predictive value independent of this overlap. The model is intended for early risk stratification at the time of admission, not for real-time dynamic prediction throughout the hospital stay. Future iterations of the model could incorporate serial measurements (e.g., repeated LVEF, biomarkers) to improve temporal precision and predictive accuracy. Until external validation is performed, the model should be used with caution and as a supplement to, not a replacement for, clinical judgment.

Additionally, our variable selection strategy included univariate screening (*p* < 0.05) as an initial step. Although this approach is common in clinical prediction modeling, it is known to carry risks of variable omission, overfitting, and biased coefficient estimates. We mitigated this by also forcing clinically important variables into the model and by conducting sensitivity analyses using alternative selection methods (stepwise regression). The consistency of the results across different methods (Table 3) suggests that any bias introduced by univariate screening is likely minimal. Nonetheless, readers should interpret the findings with this methodological consideration in mind.

## 5 Conclusion

In summary, multivariate analysis identified prior myocardial infarction, smoking, decreased left ventricular ejection fraction, elevated white blood cell count, decreased hemoglobin level, elevated platelet count, and comorbid atrial fibrillation as independent risk factors for in-hospital HF following PCI in patients with T2DM and STEMI. The nomogram prediction model based on these factors demonstrates good predictive performance and internal robustness. However, due to the lack of external validation, the model should currently be considered a preliminary tool rather than a clinically ready instrument. It may serve as a useful aid for early risk assessment in settings similar to our center, but external validation in independent, multi-center cohorts is essential before it can be recommended for widespread clinical use. Future prospective studies are needed to validate, refine, and potentially integrate this model into clinical decision-support systems.

## Data Availability

The raw data supporting the conclusions of this article will be made available by the authors, without undue reservation.
