# Supplementary material for: Analysis of risk factors for heart failure in patients with type 2 diabetes mellitus and acute ST-segment elevation myocardial infarction after percutaneous coronary intervention
Source: Front Med (Lausanne). 2026 May 8;13:1796825. doi: 10.3389/fmed.2026.1796825 (PMC13193867; doi:10.3389/fmed.2026.1796825)
Supplement: Supplementary file 1 [file Table_1.DOCX]

***Supplementary Table 1. Missing Data Proportions for Candidate Predictors in the Study Cohort (N=362)***

| Variable | Number of Missing (n) | Missing Proportion (%) |
| --- | --- | --- |
| Age (years) | 0 | 0% |
| Gender | 0 | 0% |
| Prior Myocardial Infarction | 0 | 0% |
| Smoking History | 0 | 0% |
| Left Ventricular Ejection Fraction (LVEF, %) | 15 | 4.1% |
| White Blood Cell Count (×10⁹/L) | 5 | 1.4% |
| Hemoglobin (g/L) | 6 | 1.7% |
| Platelet Count (×10⁹/L) | 10 | 2.8% |
| Atrial Fibrillation | 0 | 0% |

***Note: No variable exceeded 5% missingness. Multiple imputation was performed under the missing at random (MAR) assumption.***
